# Supplementary material for: Host-Induced Gene Silencing of an Adenylate Kinase Gene Involved in Fungal Energy Metabolism Improves Plant Resistance to Verticillium dahliae
Source: Biomolecules. 2020 Jan 12;10(1):127. doi: 10.3390/biom10010127 (PMC7023357; doi:10.3390/biom10010127)
Supplement: Supplementary file 1 [file biomolecules-10-00127-s001.pdf]

## Supplementary Materials:

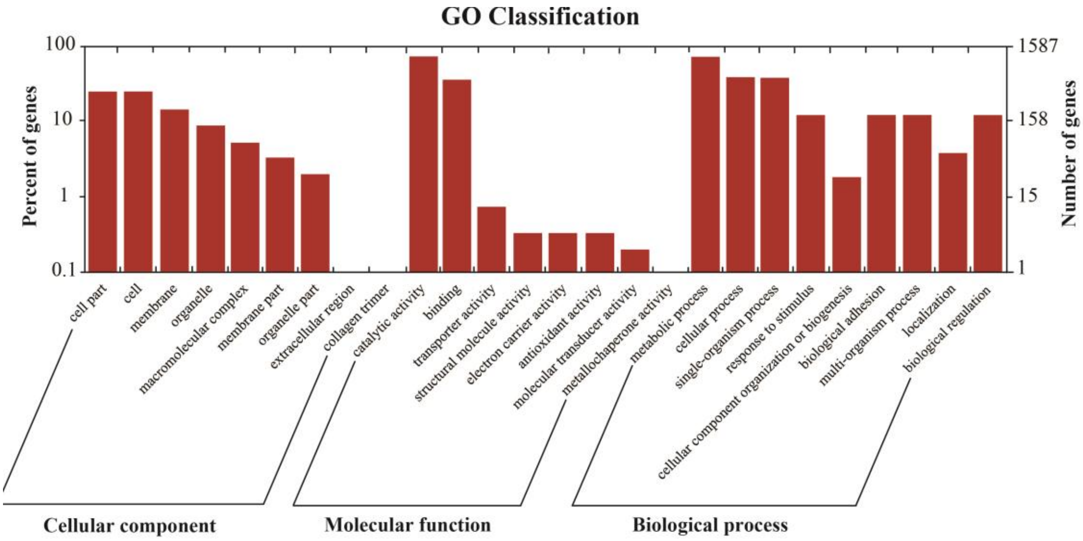


**Figure S1.** GO classification of predicted protein-encoding genes of *V. dahliae* strain VdLs.17.

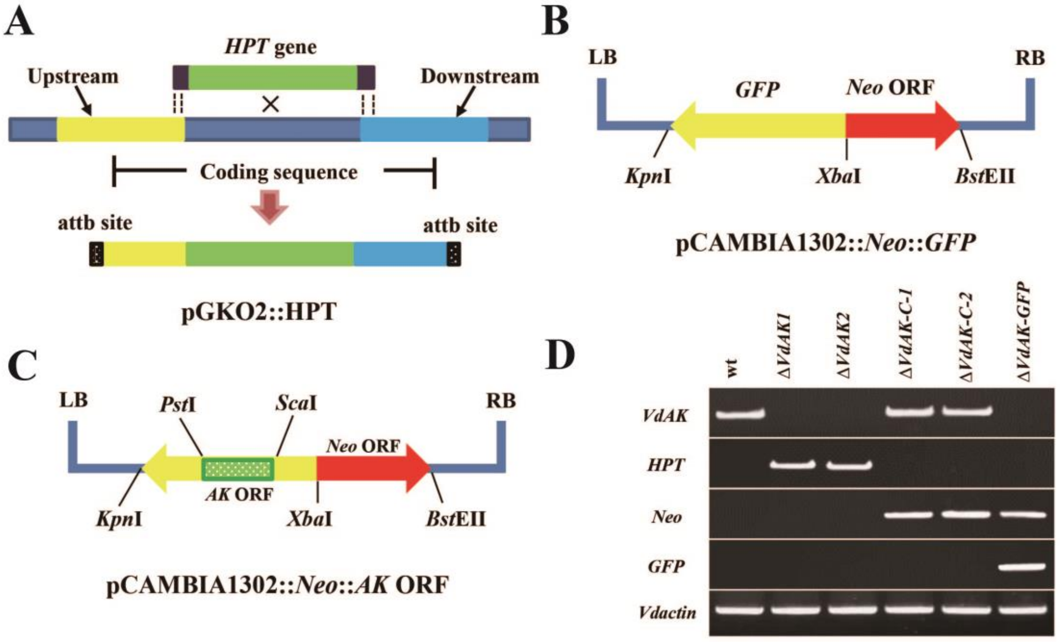


**Figure S2.** Generation of disruption (Δ*VdAK*), complementation (Δ*VdAK-C*) and Δ*VdAK-GFP* strains via homologous recombination. (A) Construction of *VdAK*-disrupted mutant Δ*VdAK*. About 1.5 kb upstream and downstream of targeted gene were fused with the hygromycin resistance cassette. The whole fragment, with attb sites, was cloned using nested PCR. (B) Construction of Δ*VdAK-GFP*. The neomycin resistance cassette, containing XbaI and BstEII restriction sites, was cloned into the pCAMBIA1302 vector. The GFP expression cassette was then cloned into the intermediate plasmid by XbaI/KpnI double-digestion (designated pCAMBIA1302::*Neo*::GFP). (C) Construction of Δ*VdAK-C*. The GFP ORF of plasmid pCAMBIA1302::*Neo*::GFP was replaced with *VdAK* ORF via ScaI/PstI double digestion

(designated pCAMBIA1302::*Neo*::*VdAK*). (D) Expression of the targeted genes was confirmed by RT-PCR.

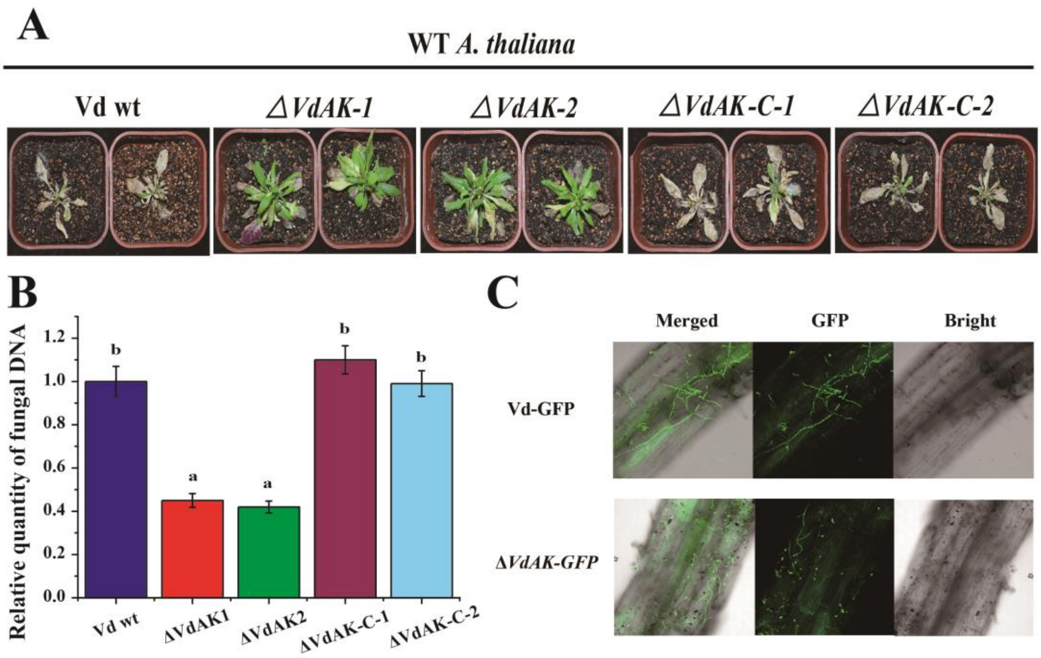


**Figure S3.** Virulence analysis of disruption (Δ*VdAK*) and complementation (Δ*VdAK-C*) mutants and wild-type *Vd* in *A. thaliana*. (A) Symptoms on seedlings at 12 days after roots were dipped in $10^6$ spores/mL of Δ*VdAK*, Δ*VdAK-C* or wild-type *Vd*. (B) Relative amounts of fungal DNA determined by RT-qPCR. Means (±SE) from three independent experiments that significantly differed among treatments in Duncan's test ($P < 0.05$) are indicated with different letters. (C) Micrographs of fluorescing fungal hyphae in *A. thaliana* root tips at 5 days post inoculation with Vd-*GFP* or Δ*VdAK-GFP*.

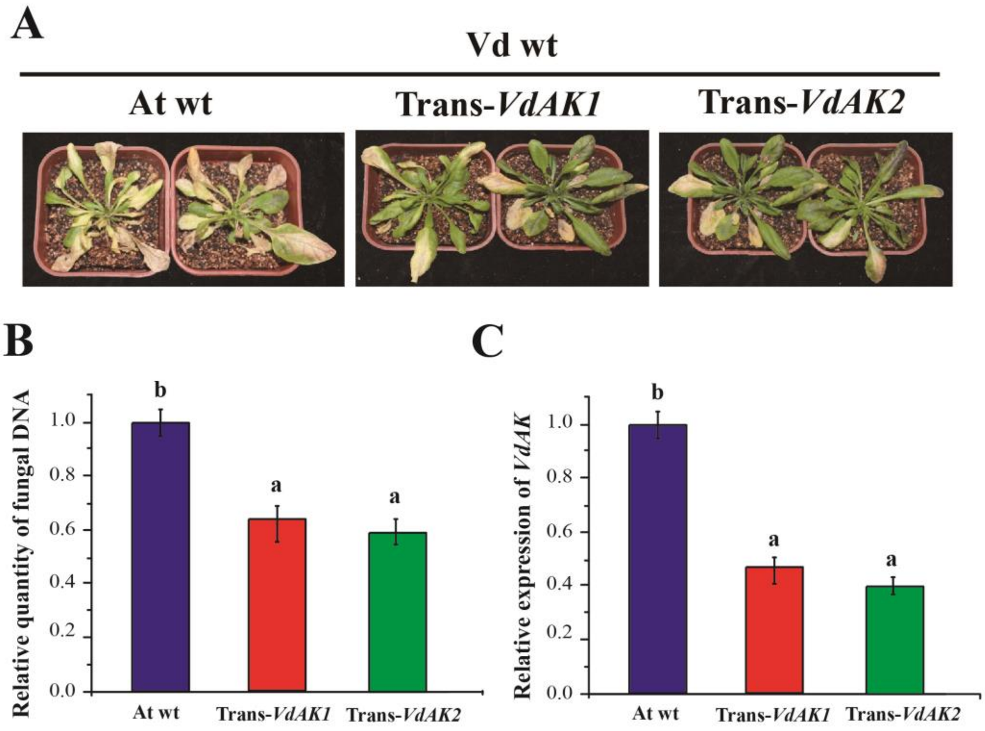


**Figure S4.** Expression of dsRNAs of *VdAK* in *A. thaliana* led to increased resistance against *Vd*. (A) Symptoms on transgenic and wild-type plants at 12 days after roots were dipped in $10^6$ spores/mL of the respective strains. (B) Relative amounts of fungal DNA determined by RT-qPCR. (C) Transcript levels of *VdAK* in stems of transgenic and wild-type plants. Means (±SE) from three independent experiments were analyzed for significant differences among treatments using Duncan's test ($P < 0.05$).

**Table S1. Information on RNAi constructs targeting 92 *V. dahliae* genes selected for HIGS and disease index from 10-12 days post inoculation of construct-treated plants**

| RNAi constructs | Primer sequences (5′ to 3′) | Gene IDs | Annotations | Disease index 10 dpi | Disease index 11 dpi | Disease index 12 dpi |
|---|---|---|---|---|---|---|
| TRV:00 | | | | 100 | 100 | 100 |
| 1 | ATAGAATTCCGTTATCCTCGTCGGTGC ATAGGATCCAGACACGGCCCGAG | VDAG_01040.1 | Adenylate kinase | 15 | 20 | 25 |
| 2 | ATAGAATTCCCATTCAGACTGCCAG ATAGGATCCCTCCTCCAAAGATCC | VDAG_05976.1 | Alpha-amylase A type-1/2 | 15 | 55 | 65 |
| 3 | ATAGAATTCATGGCGCAGAACCCC GCGTATGGAAATCCTCTCGTCCC | VDAG_07833.1 | Ankyrin repeat-containing protein | 60 | 80 | 90 |

| | | | | | | |
|---|---|---|---|---|---|---|
| 4 | ATAGAATTCTTGTCCTCTCCAACG<br>GGTGTAGATGAAGACATCGTACAAG | VDAG_07739.1 | Aquaporin-3 | 18 | 20 | 24 |
| 5 | AGTGAATTCGTGCTTCAAGCGTAC<br>AGAGGATCCCCCTTGAAGAGAGAC | VDAG_07535.1 | ADP, ATP carrier protein | 20 | 30 | 35 |
| 6 | AGAGAATTCATGGATTACCAGGGTTC<br>TGAGGATCCCACTTGTAGCAGATC | VDAG_00201.1 | Cellular nucleic acid-binding protein | 20 | 55 | 70 |
| 7 | ATAGAATTCATGTCGGAGAACCTCGC<br>ATAGGATCCGACAGTCAAGATGCC | VDAG_02332.1 | Aconitate hydratase | 20 | 45 | 70 |
| 8 | ATAGAATTCGGTCATCGAGCCTGG<br>ATATGGATCCGGAATCGTGTTCTCC | VDAG_01206.1 | Pyruvate kinase | 20 | 40 | 55 |
| 9 | AGAGAATTCTCGTCATCCGCAGTG<br>ATAGGATCCCTTGTGGCTCCTCTG | VDAG_09094.1 | Peroxisomal membrane protein PEX30 | 30 | 40 | 45 |
| 10 | ATAGAATTCACCGGAGGACTCGGG<br>ATAGGATCCCCTTGATCGTCCAGC | VDAG_03613.1 | NADPH dehydrogenase | 25 | 50 | 65 |
| 11 | AGAGAATTCTGCTAACAAGGCCGGC<br>AGAGGATCCAGTGGATGACCGAGG | VDAG_04294.1 | 60S Acidic ribosomal protein P0 | 40 | 55 | 75 |
| 12 | ATAGAATTCCAATCGACGGCATCC<br>ATAGGATCCTGCAATCGATGCTCC | VDAG_01123.1 | Hard-surface induced protein | 35 | 50 | 75 |
| 13 | AGAGAATTCCAACCTCTACGACGCC<br>ATAGGATCCCACGGAGCTTATCGG | VDAG_01774.1 | Malate synthase | 60 | 70 | 80 |
| 14 | ATAGAATTCATCAAGGCGCTCAAGC<br>AGAGGATCCAGTGAGCATGAGACG | VDAG_03232.1 | Oligosaccharyl transferase STT3 subunit | 10 | 25 | 35 |

| | | | | | | |
|---|---|---|---|---|---|---|
| 15 | ATAGAATTCGTGCCGAC ATCGACC ATAGGATCCGCCCACTG AATAGAGC | VDAG_08 270.1 | Transport protein BOS1 | 75 | 90 | 100 |
| 16 | ATAGAATTCGACACGGA GTACCTCCG AGAGGATCCTGCTGGTG ATATGCC | VDAG_05 521.1 | Vacuolar protein sorting-associated protein | 50 | 75 | 90 |
| 17 | ATAGAATTCATCAAGCG ACCGAGAGG ATAGGATCCATGCTCCCT GTCAGC | VDAG_01 656.1 | Viral A-type inclusion protein repeat protein | 25 | 60 | 75 |
| 18 | ATAGAATTCGAGCCAGC CCAACAG AGAGGATCCTGGACAAG GTGTACTCG | VDAG_06 474.1 | cAMP-dependent protein kinase | 45 | 65 | 75 |
| 19 | ATAGAATTCCACCTTCCA GGACTCC ATAGGATCCTCTCTTCTG GGTACCG | VDAG_06 474.1 | cAMP-dependent protein kinase | 80 | 85 | 95 |
| 20 | AGAGAATTCGCAGGCTA CATCAGCG ATAGGATCCGTGCTCAG CTCGTACG | VDAG_02 311.1 | Solute carrier family 25 member 45 | 50 | 70 | 85 |
| 21 | AGAGAATTCCTGGAAAG GCACCTGC ATAGGATCCAGGCATGA GAACGCC | VDAG_00 714.1 | Argininosuccinate lyase | 20 | 25 | 30 |
| 22 | TGTGAATTCGGCCTCGA GTATAAGG ATAGGATCCGCCTTCAC GTACTCG | VDAG_00 317.1 | Cryptochrome DASH | 65 | 75 | 85 |
| 23 | AGAGAATTCGTACGTCC GCTCCTGC ATAGGATCCGAGATCTG GGTCACGG | VDAG_05 375.1 | Covalently-linked cell wall protein | 70 | 80 | 95 |
| 24 | ATAGAATTCCAACGTGA CTCCTGGC AGTGGATCCTTAACACT CGTCCGTC | VDAG_10 495.1 | Endoglucanase B | 65 | 75 | 85 |
| 25 | ATAGAATTCAACGCTGT CTACTTCACG ATAGGATCCTGAGGTAG TAGTTGACG | VDAG_04 416.1 | Chitinase A | 60 | 70 | 90 |

| | | | | | | |
|---|---|---|---|---|---|---|
| 26 | AGAGAATTCGGAGCATACTGACGTCC ATAGGATCCTGTGCACAGACTGATGC | VDAG_09689.1 | Guanine nucleotide-binding protein subunit beta | 55 | 65 | 80 |
| 27 | AGAGAATTCGCCTACCAGGAAGACC ATAGGATCCGGACTTGATGTAGGCC | VDAG_07192.1 | 1,3-Beta-glucanosyltransferase gel4 | 55 | 65 | 80 |
| 28 | ATAGAATTCCACGCTCAACACGACC TTAGGATCCCTCCATGGTGTACTCG | VDAG_02243.1 | 1,3-Beta-glucanosyltransferase gel1 | 55 | 75 | 85 |
| 29 | ATAGAATTCCTTGGCCTTAGCTGGC TTAGGATCCTCTAGGGTTGAGCCG | VDAG_02071.1 | Glucosyltransferase H | 60 | 70 | 85 |
| 30 | TGAGAATTCTACAGCCAGCTCTCGC ATAGGATCCCGCTGGATATCGAGC | VDAG_06369.1 | GTP-binding protein GTR2 | 40 | 50 | 65 |
| 31 | AGAGAATTCCTGAGGACTCAGCAGGC ATAGGATCCCGGATGTGCTTCAGG | VDAG_10151.1 | NADH-ubiquinone oxidoreductase 21 kDa subunit | 60 | 70 | 80 |
| 32 | TGAGAATTCACAGTCGAGCTCGACG ATAGGATCCGGCTGAGAGAACAGGC | VDAG_04456.1 | Nuclear pore glycoprotein p62 | 50 | 60 | 70 |
| 33 | TTAGAATTCCGACCACAGCATCACG ATAGGATCCGGATCATCTCAGTGCG | VDAG_03673.1 | Nuclear distribution protein nudE | 20 | 30 | 35 |
| 34 | TGAGAATTCTCTTCTCCAACGTCGG AGAGGATCCTAATGTTGGACATGCG | VDAG_09326.1 | GTP-binding protein GTR1 | 40 | 45 | 55 |
| 35 | AGAGAATTCCAACCGTCTGGCTACC TTAGGATCCCAATGATCAGAGGGC | VDAG_09877.1 | Aspartyl/glutamyl-tRNA (Asn/Gln) amidotransferase subunit B | 50 | 65 | 75 |
| 36 | TTAGAATTCGAGCAGGATTACGTGGC ATAGGATCCATACGAGCCTGCACG | VDAG_02981.1 | Methyltransferase domain-containing protein | 45 | 55 | 65 |

| | | | | | | |
|---|---|---|---|---|---|---|
| 37 | ATAGAATTCCAAGCGGAGACTGATGG TTAGGATCCTGAATGTGCAGGTGG | VDAG_05486.1 | Atrial gland-specific antigen | 31.25 | 56.25 | 62.5 |
| 38 | TGTGAATTCTCTCCTACTCCGTCCG ATAGGATCCGGATATGTCGAGGTCTG | VDAG_00647.1 | Inner centromere protein | 6.25 | 12.5 | 18.75 |
| 39 | AGAGAATTCCTCTTATCTGTCGGCGG ATAGGATCCGTTGACGTGAGTCCG | VDAG_00787.1 | Hypothetical protein | 43.75 | 50 | 56.25 |
| 40 | ATAGAATTCATCGGGCCAAGGTGG TTAGGATCCTCACTCAGCATGCTCG | VDAG_06790.1 | PSP1 domain-containing protein | 37.5 | 43.75 | 56.25 |
| 41 | AGAGAATTCTCGCCGAGATGCTCG ATAGGATCCGGCGTCCACATTATGG | VDAG_08982.1 | Mitogen-activated protein kinase HOG1 | 68.75 | 81.25 | 87.5 |
| 42 | ATAGAATTCGACGAGATTGACGAGG ATAGGATCCTCTCAAGCTCATCAGC | VDAG_03613.1 | NADPH dehydrogenase | 25 | 31.25 | 37.5 |
| 43 | AGAGAATTCAGCTCTGGGATCAAGG ATAGGATCCTTGGTCTCGTCCTCTG | VDAG_04020.1 | NADPH dehydrogenase D | 40 | 56 | 68 |
| 44 | ATAGAATTCGCAAGATCGCTCTGGC ATAGGATCCGAGGTAGCACGAGC | VDAG_08067.1 | Pectate lyase B | 50 | 56.25 | 62.5 |
| 45 | ATAGAATTCGCCTTGCTTCGTTCG TAAGGATCCCTGCGAATGTTGTACC | VDAG_05402.1 | Pectate lyase | 56.25 | 62.5 | 68.75 |
| 46 | ATAGAATTCGGACGAAGCAGACACG AGAGGATCCTCTCGTGATAGGGAGG | VDAG_08663.1 | cAMP-dependent protein kinase regulatory subunit | 50 | 55 | 69 |
| 47 | ATAGAATTCGCTTCGAGCGTCATGG ATAGGATCCGATCCCGTCATCTCG | VDAG_08656.1 | Cell pattern formation-associated protein stua | 56.25 | 68.75 | 75 |

| | | | | | | |
|---|---|---|---|---|---|---|
| 48 | AGAGAATTCCAATGGCAGCAGAGC ATAGGATCCGGTCGAGGTATAGGGC | VDAG_06649.1 | Dual specificity protein kinase YAK1 | 20 | 30 | 35 |
| 49 | TTAGAATTCGTCAACCAGCTCGAGG ATAGGATCCCTTGATCATGGCAGG | VDAG_07142.1 | Polysaccharide deacetylase family protein | 65 | 75 | 80 |
| 50 | ATAGAATTCCAGAAGCCTGTCGTGC ATAGGATCCAATCTCGTTCGTGACG | VDAG_08977.1 | ATP-dependent protease La | 25 | 40 | 45 |
| 51 | ATAGAATTCCGTCACCATCAACAAGGC ATAGGATCCACCTTGCTCGTGTTGG | VDAG_08977.1 | ATP-dependent protease La | 55 | 65 | 70 |
| 52 | ATAGAATTCGTCGAAGATACCACGGC ATAGGATCCGTAGCTCTGCGTCAGG | VDAG_00279.1 | Import receptor | 35 | 45 | 50 |
| 53 | ATAGAATTCACGTACGCTGCGTATCG TTAGGATCCTGATGGTGTACTCGGC | VDAG_05060.1 | Multidrug resistance protein | 65 | 75 | 85 |
| 54 | ATAGAATTCGTCGACCAGGAAGTCG ATAGGATCCTACGGTAAGCCGTCG | VDAG_05084.1 | ATP-dependent bile acid permease | 25 | 30 | 35 |
| 55 | ATAGAATTCCAACGGTGACCTCAAGG ATAGGATCCCTCCTCAGCCTTCTCC | VDAG_04294.1, | 60S Acidic ribosomal protein P0 | 55 | 70 | 75 |
| 56 | ATAGAATTCGGCAACCGAGATAGCC ATAGGATCCCTGGAGTACGGTAACC | VDAG_00335.1 | Secreted protein K | 60 | 65 | 70 |
| 57 | AGAGAATTCATGCTCTCCAGCCTC ATAGGATCCTCTCTCTGCAGCACG | VDAG_07788.1 | Microsomal signal peptidase 18 kDa subunit | 60 | 65 | 70 |
| 58 | AGAGAATTCGACGACATGATCTCCCAG ATAGGATCCGGTGAAGGTGACCTCC | VDAG_07546.1 | Stress protein | 55 | 65 | 75 |

| | | | | | | |
|---|---|---|---|---|---|---|
| 59 | ATAGAATTCACCTGGCC ATGAGGTC ATAGGATCCTGCGACAG CATTCAC | VDAG_03 187.1 | General stress protein | 55 | 65 | 85 |
| 60 | AGAGAATTCGCAAGACG AACGTTCC ATAGGATCCACTCGTGG AAGTGCG | VDAG_01 389.1 | Glycogen synthase | 65 | 80 | 85 |
| 61 | ATAGAATTCCCTTGGGG CTACACCC ATAGGATCCTGCTGCTGC TAGTGCC | VDAG_01 389.1 | Glycogen synthase | 55 | 70 | 90 |
| 62 | AGAGAATTCCTCGTTCAT CTACGTCG ATAGGATCCCTCTGGTA CGTACTGCC | VDAG_01 254.1 | Tryptophan synthase | 50 | 55 | 65 |
| 63 | AGAGAATTCCTGGTATCT CAAGGCG ATAGGATCCAGTCGTCG TGGTAGG | VDAG_07 000.1 | Killer toxin subunits alpha/beta | 45 | 60 | 65 |
| 64 | AGAGAATTCCTGGGACT ACAAGAGCG ATAGGATCCGAGAGGCT CACAAGC | VDAG_00 901.1 | Killer toxin subunits alpha/beta | 55 | 65 | 70 |
| 65 | AGAGAATTCTGTTCAAC AGCAAGCC AGAGGATCCTGTCAAGA ATGGCAC | VDAG_00 825.1 | Vacuolar protease A | 62 | 72 | 80 |
| 66 | GAGGAATTCAGAATACG TGGAACGGG ATAGGATCCGATGCTGC TCGTTGAC | VDAG_08 621.1 | Carbon catabolite-derepress ing protein kinase | 50 | 60 | 75 |
| 67 | ATAGAATTCCTCCAATCC GTTGTCC ATAGGATCCAGGAGCAG CTGGTC | VDAG_04 529.1 | Thioredoxin | 16 | 23 | 28 |
| 68 | AGAGAATTCAGTCTGGC TCATCACCG ATAGGATCCAGGTTGGC AACGATG | VDAG_09 532.1 | Serine 3-dehydrogenase | 60 | 75 | 85 |
| 69 | AGAGAATTCCGTCGACG GCAAGC AGAGGATCCTTGTGCCA GCTGAGG | VDAG_05 692.1 | Uricase | 70 | 85 | 95 |

| | | | | | | |
|---|---|---|---|---|---|---|
| 70 | AGAGAATTCGCAAGCGTCCAGAAG ATAGGATCCAGACCCAGGACCCTC | VDAG_09026.1 | NADH-ubiquinone oxidoreductase 29.9 kDa subunit | 25 | 30 | 35 |
| 71 | TATGAATTCGGTCGCACAGTACAACG ATAGGATCCCGACTCAATGTCGAGG | VDAG_01642.1 | Pyruvate dehydrogenase E1 component subunit beta | 70 | 90 | 95 |
| 72 | AGAGAATTCCCGAGGAGACTCTGCG ATAGGATCCGGTTGGAGCCGTACC | VDAG_03252.1 | Glycine-rich RNA-binding protein | 60 | 70 | 75 |
| 73 | ATAGAATTCCGTTCAAGAATATGCCG TATGGATCCACCGTGAGACTTGAC | VDAG_07546.1 | Stress protein | 10 | 15 | 20 |
| 74 | CTAGAATTCAAGGGCATCCTCGTTC ATAGGATCCCCAGAAGTCTTCAGCC | VDAG_06669.1 | Hypothetical protein | 70 | 85 | 90 |
| 75 | ATAGAATTCCCTCCGGCATGAAGG TTAGGATCCTGAGCACCTCCATCG | VDAG_08238.1 | Glycerate kinase | 80 | 85 | 90 |
| 76 | AGTGAATTCTGCGATTGCGACATCC ATAGGATCCCTATCCGACCCATGGC | VDAG_04980.1 | Thymidylate kinase | 60 | 70 | 85 |
| 77 | ATAGAATTCGTCATCTGAGCAGCAGC ATAGGATCCGCATTGTAGCTCATGG | VDAG_01726.1 | Choline kinase | 70 | 90 | 95 |
| 78 | ATAGAATTCCGGCACCACGGTCAT ATAGGATCCCGAGGACACGAGCAC | VDAG_02259 | Glutamate decarboxylase | 65 | 75 | 95 |
| 79 | ATAGAATTCGTGTTGGTTGAGTCGA ATAGGATCCAACAGCCCCGTGCCGA | VDAG_06992 | Ferric reductase transmembrane component 4 | 60 | 70 | 85 |
| 80 | ATAGAATTCTCCGCTCCTGCGTCCC ATAGGATCCGATCTGGGTCACGGC | VDAG_04380 | Cytochrome P450 51 | 65 | 75 | 85 |

| | | | | | | |
|---|---|---|---|---|---|---|
| 81 | ATAGAATTCATTCCAACGTGACTC ATAGGATCCCTTAACACTCGTCCG | VDAG_10495 | Endoglucanase B | 30 | 35 | 40 |
| 82 | ATAGAATTCTCGAGCCAGCCCAAC ATAGGATCCCTGGACAAGGTGTA | VDAG_06474 | cAMP-dependent protein kinase | 60 | 70 | 75 |
| 83 | ATAGAATTCCGTCAACCAGCTCGA ATAGGATCCCCTTGATCATGGCAGG | VDAG_07142 | Polysaccharide deacetylase family protein | 40 | 45 | 60 |
| 84 | ATAGAATTCGCTCAAGCCCTACGGCC ATAGGATCCAGGATCTGGCCGAAGCC | VDAG_01845.1 | Branched-chain-amino-acid aminotransferase | 65 | 75 | 95 |
| 85 | ATAGAATTCGTGGTGCGCGTCTACC ATAGGATCCGATGGCCAGCTCGAGG | VDBG_07510.1 | Xylulose-5-phosphate phosphoketolase | 60 | 70 | 75 |
| 86 | ATAGAATTCCCGTACTTCCGGCAAC ATAGGATCCGCGACAGCGTAACGA | VDAG_05940.1, | 60S Ribosomal protein L2 | 55 | 60 | 65 |
| 87 | ATAGAATTCGTGTTGGTTGAGTCGA ATAGGATCCAACAGATCCGTGCCG | VDAG_06992.1 | Ferric reductase transmembrane component 4 | 60 | 80 | 100 |
| 88 | ATAGAATTCCAAGTGGTACGGCACCA ATAGGATCCCAATGGTCGAGGACAC | VDAG_01692.1 | Endoglucanase | 55 | 65 | 75 |
| 89 | ATAGAATTCCACGGACTTCCTGAC ATAGGATCCAGAGACGGAGTGGGT | VDBG_00534.1 | 1,3-Beta-glucanosyltransferase gel1 | 70 | 80 | 90 |
| 90 | ATAGAATTCGAGACCGAGAAGTCG ATAGGATCCACAGAGCGGGCGGGG | VDBG_07986.1 | PH domain-containing protein | 65 | 70 | 80 |
| 91 | ATAGAATTCGCACATTGTCGCTCTCG ATAGGATCCCAGGTAGTGGCTCAAGG | VDAG_09595.1 | Chitin synthase export chaperone | 55 | 64 | 78 |

| | | | | | | |
|---|---|---|---|---|---|---|
| 92 | ATAGAATTCGGCCTCGAGTATAAGGA<br>ATAGGATCCGCCTTCACGTACTCGC | VDAG_00317.1 | Cryptochrome DASH | 50 | 70 | 85 |

**Table S2. Primers used to produce the *VdAK* RNAi construct**

| Primer | Sequences (5′ to 3′) |
|---|---|
| trans-VdAK | AAAAAAGCAGGCTCGTTATCCTCGTCGGTGC<br>AAGAAAGCTGGGTAGACACGGCCCGAA |
| BP site | GGGGACAAGTTTGTACAAAAAAGCAGGCT<br>GGGGACCACTTTGTACAAGAAAGCTGGGT |
| Det-VdAK | ACTCGCCTAACTGTGCCTCTA<br>GGAACCCATCCAGAATGAAGG |

**Table S3. Primers used to construct mutant strains of *V. dahliae***

| **Primer** | **Sequences (5′ to 3′)** |
|---|---|
| Hygromycin resistant expression cassette | TTGAAGGAGCATTTTTGGGC<br>TTATCTTTGCGAACCCAGGG |
| ΔVdAK | 5F: CAGAGTACCCTGACGGCATCGGC<br>5R: GCCCAAAAATGCTCCTTCAAATCAAGCGCATGATAAGGTCG<br>3F: CCCTGGGTTCGCAAAGATAACTTCGACGTGATCATGCAGCGC<br>3R: GGAAGTCTCCTGGGGTGGTGGTGG<br>NEST-F: GGGGACAAGTTTGTACAAAAAAGCAGGCTCGTCATTCCATTCCACGGACCC<br>NEST-R: GGGGACCACTTTGTACAAGAAAGCTGGGTCAAGGTACAATGTCACATACGG |
| Neomycin resistant expression cassette | aat***tctaga***GTTTGCGGGCTGTCTTGACG<br>ata***ggtcacc***TACCTGTGCATTCTGGGTAA |
| gGFP expression cassette | ggc***tctaga***CTTTCGACACTGAAATACGTCG<br>ata***ggtacc***GCATCAGAGCAGATTGTACTGAGAG |
| ΔVdAK-com | aaa***agtact***ATGAGACTTCGAAAGGCAG<br>aaa***ctgcag***TCAAGCCGCAAAGCGTTT |
| Det-HPH | TTCGACAGCGTCTCCGACCTGA |

| | |
|---|---|
| | AGATGTTGGCGACCTCGTATTGGG |
| Det-ΔVdAK | TATACAAGAATGGTTGGCTGCGTCCC<br>GTGGATGGAAGGTGGTATTGTAGACACG |
| Det-neo | GTTGTCACTGAAGCGGGAAGGG<br>GCGATACCGTAAAGCACGAGGAA |
| Det-GFP | CGACGTAAACGGCCACAAGTT<br>TCTTTGCTCAGGGCGGACTGG |

Note: Restriction sites are in bold italic font.

**Table S4. Primers used for RT-qPCR**

| Primer | Sequences (5′ to 3′) |
|---|---|
| qRT-VdAK | GTGTCTACAATACCACCTTCCA<br>CTCATCACTGCTCATACCCTC |
| qRT-VA | GGGTATTCAGACCCTATTGGACG<br>CGAACTTCTTGTACTCAGCCTCC |
| qRT-VdATP6 | CTAGACCAATTTGAAATAAGA<br>AAAGATTCTTGGCTAATAGAT |
| qRT-VdAC | TCTCCATCGTCTTCACCGACATCA<br>TCTGCACGGCGAAACACCACA |
| qRT-VdATP-PRT | CGACGCCAACGTGCGGTCCTACAA<br>GCCCGAGAAGCTCGTGCCAAT |
| qRT-Vdactin | GGCTTCCTCAAGGTCGGCTATG<br>GCTGCATGTCATCCCACTTCTTC |
| qRT-VdITS | CCGCCGGTCCATCAGTCTCTCTGTTTATAC<br>CGCCTGCGGGACTCCGATGCGAGCTGTAAC |
| RT-Nbactin | GGACCTTTATGGAAACATTGTGCTCAGT<br>CCAAGATAGAACCTCCAATCCAGACAC |
| RT-AtEF-1$\alpha$ | TGAGCACGCTCTTCTTGCTTTC<br>GGTGGTGGCATCCATCTTGTTA |

**Table S5. Information on sRNAs from *V. dahliae* isolated from *Vd*-infected seedlings**

| Sample | Total sRNAs | Mapped sRNAs | Total mapped sRNAs |
|---|---|---|---|
| Col_1 | 10786798 (100.00 %) | 6919974 (64.15 %) | 6650 (0.10 %) |
| RNAi_1 | 4068549 (100.00 %) | 3039943 (74.72 %) | 5015 (0.16 %) |
| RNAi_2 | 8235986 (100.00 %) | 5599526 (67.99 %) | 5090 (0.09 %) |
| RNAi_3 | 9820717 (100.00 %) | 6890613 (70.1 %) | 9929 (0.14 %) |

**Table S6. Sixteen *Vd* candidate pathogenicity/virulence factor genes identified by HIGS**

| Gene ID | Annotation | Disease index | | |
|---|---|---|---|---|
| | | **10 dpi** | **11 dpi** | **12 dpi** |
| VDAG_01040.1 | Adenylate kinase | 15 | 20 | 25 |
| VDAG_07535.1 | ADP, ATP carrier protein | 20 | 30 | 35 |
| VDAG_01206.1 | Pyruvate kinase | 20 | 40 | 55 |
| VDAG_09094.1 | Peroxisomal membrane protein PEX30 | 30 | 40 | 45 |
| VDAG_03232.1 | Oligosaccharyl transferase STT3 subunit | 10 | 25 | 35 |
| VDAG_00714.1 | Argininosuccinate lyase | 20 | 25 | 30 |
| VDAG_03673.1 | Nuclear distribution protein nudE | 20 | 30 | 35 |
| VDAG_00647.1 | Inner centromere protein | 6.25 | 12.5 | 18.75 |
| VDAG_03613.1 | NADPH dehydrogenase | 25 | 31.25 | 37.5 |
| VDAG_06649.1 | Dual specificity protein kinase YAK1 | 20 | 30 | 35 |
| VDAG_08977.1 | ATP-dependent protease La | 25 | 40 | 45 |
| VDAG_05084.1 | ATP-dependent bile acid permease | 25 | 30 | 35 |
| VDAG_04529.1 | Thioredoxin | 16 | 23 | 28 |
| VDAG_09026.1 | NADH ubiquinone oxidoreductase | 25 | 30 | 35 |
| VDAG_07546.1 | Stress protein | 10 | 15 | 20 |
| VDAG_10495.1 | Endoglucanase B | 30 | 35 | 40 |
| Control | | 100 | 100 | 100 |
